# Vitamins and Uterine Fibroids: Current Data on Pathophysiology and Possible Clinical Relevance

**DOI:** 10.3390/ijms21155528

**Published:** 2020-08-01

**Authors:** Michał Ciebiera, Mohamed Ali, Magdalena Zgliczyńska, Maciej Skrzypczak, Ayman Al-Hendy

**Affiliations:** 1 Second Department of Obstetrics and Gynecology, The Center of Postgraduate Medical Education, 01-809 Warsaw, Poland; zgliczynska.magda@gmail.com; 2 Department of Surgery, University of Illinois at Chicago, Chicago, IL 60612, USA; mali78@uic.edu; 3 Clinical Pharmacy Department, Faculty of Pharmacy, Ain Shams University, 11566 Cairo, Egypt; 4 Second Department of Gynecology, Medical University of Lublin, 20-954 Lublin, Poland; skrzypczakmk8@gmail.com

**Keywords:** uterine fibroid, leiomyoma, pathophysiology, vitamins, vitamin A, vitamin B3, vitamin C, vitamin D, vitamin E, vitamin K

## Abstract

Uterine fibroid (UF) is the most common benign tumor pathology of the female reproductive organs. UFs constitute the main reason for a hysterectomy and hospitalization due to gynecological conditions. UFs consist of uterine smooth muscle immersed in a large amount of extracellular matrix (ECM). Genetic studies have demonstrated that UFs are monoclonal tumors originating from the myometrial stem cells that have underwent specific molecular changes to tumor initiating stem cells which proliferate and differentiate later under the influence of steroid hormones. There is growing interest in the role of micronutrients, for example, vitamins, in UFs. This article is a comprehensive review of publications regarding the available data concerning the role of vitamins in the biology and management of UFs. In summary, the results showed that some vitamins are important in the biology and pathophysiology of UFs. For example, vitamins A and D deserve particular attention following studies of their influence on the treatment of UF tumors. Vitamins B3, C, and E have not been as widely studied as the abovementioned vitamins. However, more research could reveal their potential role in UF biology.

## 1. Introduction

### 1.1. Uterine Fibroids: Epidemiology and Symptomatology

Uterine fibroid (UF) is the most common benign tumor occurring in women of reproductive age. The name reflects its hard and fibrous consistency, in addition to its characteristic microscopic presentation [1,2]. UF represents a localized proliferation of myometrial smooth muscle cells surrounded by a pseudocapsule of compressed muscle fibers. These tumors are usually detected incidentally during routine gynecological check-ups through a bimanual pelvic or ultrasound examination [1].

The occurrence of UF, in women, is estimated to be from approximately 25% up to as much as 70–80%, depending on several risk factors and the population [1,3,4]. Ethnicity and age are the most important confirmed risk factors of UF development [4]. Other factors include age at menarche, parity, environmental exposure, inappropriate diet, and lifestyle [1,5,6]. A large proportion of UF patients present with clinical manifestations that disrupt normal functioning and result in gynecological appointments [7]. UF-triggered manifestations can be grossly divided into three main groups, i.e., symptoms associated with abnormal bleeding from the reproductive tract, symptoms related to the presence of a pathological mass in the minor pelvic cavity or abdominal cavity, and symptoms related to reproductive dysfunction [1,7]. Numerous treatment modalities are available ranging from noninvasive treatments which use drugs or devices based on ultrasound or other physical methods for minimally invasive procedures of lesion removal, through to open surgery resulting in hysterectomy [8]. The size and location of the lesion are the main factors which particularly influence symptom severity and the necessity for suitable treatment. Other important determinants of treatment selection include the patient’s age and reproductive plans, the gynecologist’s skills, and the availability of appropriate medical devices [9,10]. UFs constitute the main reason for hysterectomies and hospitalization due to gynecological conditions in the United States [11]. Moreover, the financial burden in United States due to UF has been estimated to have reached USD 34 billion, including direct costs such as the cost of medications, medical personnel salaries, and the cost of surgery, as well as indirect costs related to missing work, subsequent gynecological appointments, and diagnostics [12]. These factors [12] have increased interest in gaining a better understanding of UF biology and searching for new treatment methods [13,14].

### 1.2. Biology of Uterine Fibroids: Overview

UFs consist of uterine smooth muscle immersed in a large amount of disrupted and disorganized extracellular matrix (ECM) [15,16]. Genetic research has demonstrated that UFs are monoclonal tumors originating from the myometrial stem cells which have undergone specific molecular changes [17,18]. An important study by Mäkinen et al., conducted in 2011, showed specific mutations within the gene coding the mediator complex subunit 12 (MED12), in 70% of studied UFs. All *MED12* gene mutations were located within exon 2 and, most probably, they presented the strongest association with UF development [18]. Various studies were conducted in the following years and confirmed these observations [19,20].

According to the majority of published studies, UF growth is largely dependent on steroid sex hormones [21,22]. It has been verified by the fact that UFs do not develop before menarche and their size decreases after menopause. Moreover, UFs are more common in obese women, as the excess fat tissue can generate more steroid hormones [1,21]. Current data have shown that the use of gonadotropin-releasing hormone (GnRH) agonists induced a menopause-like condition, and reduced the size of UFs [23,24]. Interestingly, when GnRH agonist preparations were discontinued, UFs regrew and became symptomatic in the majority of cases [24].

The conversion of a normal myometrial stem cell into a tumor initiating stem cell with a tendency towards abnormal growth is the first link of the complex UF pathogenesis chain. Subsequently, it further divides and the tumor grows [22]. The microenvironment imparts stimulation effects towards such a division and ECM production [16], including steroid hormones which can be responsible for the expression of genes, growth factors, and cytokines [25,26].

Growth factors are a group of polypeptide ligands which play major biological roles including proliferation, differentiation, angiogenesis, inflammation, and fibrosis. They act through different signaling pathways, including Smad proteins, extracellular signal-regulated kinases (ERKs), phosphoinositide 3-kinase (PI3K), and wingless type (Wnt)/β-catenin, and they regulate major cellular processes which are linked to UF development and growth [27]. Several therapeutic compounds can modulate growth factors and, subsequently, their signaling pathways, which results in tumor volume reduction and the alleviation of symptoms [25,28]. Transforming growth factor β (TGF-β), and three of its isoforms in particular, seem to be highly involved in the pathophysiology of UFs [26], in addition to other factors, including tumor necrosis factor α (TNF-α) [29], vascular endothelial growth factor (VEGF), and fibroblast growth factors (FGFs) [25].

### 1.3. Diet, Healthy Lifestyle, and Uterine Fibroids: An Overview

The positive impact of an active health-promoting lifestyle on the development of UFs is a trendy subject to explore. Exposure to different environmental and nutritional factors, such as plastic products, cosmetics, and other chemicals, as well as the intake of soybean milk, food additives, sweeteners, and preserved foods can increase the risk of UFs [4,30]. Furthermore, the incidence of UFs has been shown to be greater in populations consuming more red meat, for example, beef [31]. The lesions have also been positively associated with the current consumption of alcohol, particularly beer [32,33], whereas a diet rich in green vegetables and citrus fruits seemed to be a protective factor [34]. Many people believe in the beneficial power of vitamins, both in isolated diseases, and for health in general. However, in many cases, the data are questionable and lack scientific confirmation. Therefore, it is important to depend on reliable sources [35]. In a study by Wise et al. (2011), the intake of fruit and vegetable was inversely associated with UFs. The authors concluded that dietary intake of fruit and vitamins was of great importance against UFs [34]. Similarly, He et al. (2013) showed that vegetable and fruit intake was a protective factor, whereas high body mass index (BMI) due to unhealthy diet could increase the risk of UFs in premenopausal women [6]. Milk and dairy can also help to reduce UFs [36]. To conclude, healthy diet, based on vegetables and multigrain products, and the optimal intensity of physical exercise can significantly relieve the burden of UF and ease its symptoms [6,37]. According to the available data, diet changes and some natural compounds help to protect against UF and to relieve the symptoms [38,39].

### 1.4. Vitamins and Uterine Fibroids: Introduction

Vitamins have been used in medicine for many decades. Some of them are used for treatment, for example, vitamin K in neonates [40] or vitamin A in the treatment of dermal lesions [41]. Since there has been a recent distinct trend towards noninvasive or minimally invasive procedures for UF treatment [7], including various new compounds (hormonal and nonhormonal) [38,42], numerous authors have suggested the beneficial impact of a suitable diet and supplements on the regulation of UF development risk and growth [39,43]. Data regarding vitamin D utility in UFs are generally accessible and more widely recognized [44], while knowledge about other vitamins and their anti-UF potential role is practically negligible.

Therefore, the aim of this review was to summarize all available data regarding their known and hypothetical role in the biology and pathophysiology of UFs, clinical implications, as well as possible future directions.

## 2. Methodology

This article is a comprehensive review of all publications discussing the current role of vitamins in the biology and management of UFs. The article includes both basic science and available clinical data. A literature search was conducted in PubMed of the National Library of Medicine using the following key words: “uterine fibroid”, “leiomyoma”, “vitamins”, “vitamin A”, “vitamin B”, “vitamin C”, “vitamin D”, “vitamin E”, and “vitamin K”. During our search, we combined the keywords into pairs. Databases were extensively searched for all original and review articles/book chapters published in English, up to May 2020. In addition, articles from the reference sections of the reviewed articles were searched. The above search resulted in retrieving literature concerning vitamins A, B3, C, D, E, and K in the field of UFs and the data are discussed in the present manuscript.

## 3. Discussion

This section includes a detailed analysis of vitamins which are correlated, or may be correlated, with the biological processes in UFs, or can be used in UF prevention and treatment according to currently available literature.

### 3.1. Vitamin A and Uterine Fibroids

Vitamin A is a fat-soluble vitamin that plays numerous roles throughout the human body. It is involved in the process of vision via its cooperation in the production of visual pigment [45,46]. It also exerts a significant impact on cellular differentiation, gene expression, and immunity. According to some experts, it also influences the regulation of osseous tissue cell activity, as well as the appropriate growth and development of bones [47]. Moreover, it is involved in the epithelial function of the skin, digestive tract, or the respiratory system [46]. It has antioxidant potency and presents moderate antineoplastic activity. Vitamin A deficiency can cause visual disorders, disrupted skin function, and an increased risk of infection [48]. Conversely, an excessive level of vitamin A can be toxic and lead to death. In the case of a pregnancy, vitamin A excess may be teratogenic and harmful for the fetus [49].

The abovementioned role of vitamin A in cellular differentiation, gene expression, and immunity provided the basis for research on its participation in processes important for UFs. The interest in this area has been observed since 1980 [50]. Martin et al. (2011) showed a statistically significant dose-response inverse relationship between vitamin A and UFs, in a study performed in 887 women aged 20–49. The findings did not change, even after statistical adjustment for different traits including the age, race or education, BMI, and oral contraception use. The authors concluded that it was possible that certain micronutrients (including vitamin A) affected UF development [51]. Wise et al. (2011) demonstrated a correlation between vitamin A obtained from the diet and the occurrence of UFs. In this study, the authors followed 22,583 premenopausal women. Interestingly, the inverse association for dietary vitamin A appeared to be connected with preformed vitamin A (from animal sources) and not with provitamin A (from fruits and vegetables). Additionally, fruit and vegetable intake was inversely associated with UF and the association was stronger for fruit when served two or more times per day. The authors of this study concluded that the risk of UFs among women with a greater dietary intake of fruit and preformed vitamin A was lower [34].

Moreover, recent data confirmed the important role of vitamin A in the pathological signal transduction in UFs. All-trans retinoic acid (more commonly known as retinoic acid (RA)), is an active metabolite of vitamin A that mediates its growth-related functions [52]. RA receptors act as heterodimers with retinoid X receptors (RXRs) which act as heterodimers for several other nuclear receptors, including the vitamin D receptor (VDR) and the peroxisome proliferator-activated receptor (PPAR) [53]. Current studies have shown an antiproliferative effect on UFs [54]. Catherino and Malik (2007) demonstrated the reduced amount of cellular RA in UFs as compared with the normal myometrium. The authors concluded that UF alterations could occur partly due to a decrease in RA exposure [55]. Similarly, Tsibris et al. (1999) showed that UFs in guinea pig models had higher levels of PPARγ and RXR-α during the follicular phase of the menstrual cycle. In the same study, a PPARγ agonist, troglitazone, was administered along with estradiol and all-trans retinoic acid, which resulted in a quick growth of large UFs, never seen in this model before [56].

Other studies have shown that UFs exhibited differential expression of various proteins, enzymes, and receptors involved in the RA molecular pathway (e.g., aldehyde dehydrogenase 1) [57,58]. Moreover, the abnormal transcriptional activity of those pathways may be associated with potential UF development [27,59]. In 2008, UF exposure to RA inhibited UF cell proliferation, ECM formation, and TGF-β expression. It was concluded that RA altered the UF phenotype [60]. Lattuada et al. (2011) showed that the accumulation of RXR in UFs was associated with a delayed ligand-dependent proteasome-mediated degradation. The researchers suggested that changes of the ubiquitin/proteasome-dependent degradation of RXR by phosphorylation could be responsible for the accumulation of the receptor, and thus the dysregulation of RA-derived genes [61]. Ben-Sasson et al. (2011) detected the effect of RA on the expression and activation of the PI3K/Akt pathways. The results confirmed the occurrence of connections between RA and PI3K/Akt pathways, whose specific signaling products influence the outcome of UF growth by regulating cell proliferation and apoptosis [62].

Although, collectively, results suggest a promising role of RXR in UFs, the findings are not conclusive due to the limitations of the studies. The RXR ligand (LGD1069) appeared to exert a positive effect on UF size in animal models [63]. According to the authors, the reduction observed in most studied UF tumors, in treated animals, was mediated by a significant increase in the level of apoptosis rather than a decrease in UF cell proliferation [63]. A recent study showed that liarozole (a retinoic acid metabolism-blocking agent) inhibited ECM production in immortalized UF cells, whereas normal myometrial cells were not affected by this agent [64]. In a study by Friedman et al. (2016), treatment with vitamin A showed an effect on a UF-derived PI3K pathway and, subsequently, affected UF cell growth [65]. More recently, Heinonen et al. (2017) studied a global metabolomic profiling of UFs. Interestingly, UF lesions displayed a reduction of specific vitamin and various cofactor metabolites, which suggested alterations in redox state signaling and enzyme functions. In the described study, a total of 641 metabolites were detected and all UFs displayed reduced homocarnosine and heme metabolite levels. In the case of vitamin A, the authors reported a significant expression reduction in UFs with *MED12* gene mutations and triple wild-type subtypes of UFs. This study underlined the importance of heme and polyamine metabolism and validated the dysregulation of vitamin A in primary UF tissues [66]. In 2016, the same group showed that gene expression data also revealed RA receptor activation as one of the most significantly dysregulated pathways in UF tumors. The authors suggested that reduced vitamin A levels could reflect the increased rate of RA metabolism in UFs [67].

All these findings suggest vitamin A-derived pathway dysregulation in UF tissues and support the need for more studies to search for possible therapeutic candidates. In our viewpoint, it would be particularly important to research possible vitamin A deficiency as the cause of UF occurrence or its possible supplementation as a solitary agent, or in combination with other substances, in an attempt to treat or provide the prophylaxis of UFs.

### 3.2. Vitamin B3 and Uterine Fibroids

Vitamin B3 (a combination of nicotinic acid and nicotinamide) is an organic compound and acts as a coenzyme to dehydrogenase enzymes in the transfer of the hydride. It is an essential component of the electron carriers, i.e., nicotinamide adenine dinucleotide (NAD) and nicotinamide adenine dinucleotide phosphate (NADP) [68]. Reduced intracellular NAD concentrations lead to the inhibition of cell division and growth which can finally increase aging and cell death [69]. NAD-dependent enzymes, such as sirtuin proteins, are known for their genome protection and repair activities. Therefore, some cancers occur due to their decreased activity [69,70]. Currently, several human sirtuin isoforms are considered to be attractive therapeutic targets for aging-related, inflammatory, and neurodegenerative disorders [71]. They also show an effect on controlling fibrotic-related processes and are considered to be a primary positive mediator of endothelial-mesenchymal transition [72]. Vitamin B3 deficiency can cause pellagra with signs and symptoms including skin and mouth lesions, anemia, headaches, and tiredness [73].

Next, we discuss some facts and hypotheses concerning the role of niacin in UF biology. Niacin presents anti-inflammatory effects in a variety of tissues including the nervous system, gastrointestinal tract, and blood vessels through the activation of niacin receptor 1 (NIACR1) [74,75], whereas nicotinamide does not activate NIACR1. Conversely, both niacin and nicotinamide activate the G protein-coupled estrogen receptor (GPER) [76]. Santolla et al. (2014) stated that nicotinic acid and nicotinamide bound to and activated GPER-mediated signaling in breast cancer cells (which depends on steroid hormones similar to UFs). The study showed that the two molecules were able to upregulate GPER-derived genes through the ERK transduction pathway. The same group also discovered that nicotinic acid was involved in inflammatory processes where it upregulated intercellular adhesion molecule 1 triggered by TNF-α and stimulated the formation of endothelial tubes [76]. Furthermore, it was demonstrated, in 1998, that a combined treatment with taurine and niacin ameliorated bleomycin-induced lung fibrosis, due to the inhibition of TGF-β1 messenger RNA (mRNA) expression [77]. In a more recent study by Arauz et al. (2015), the authors also found that vitamin B3 was connected with fibrosis and ECM accumulation. They showed that nicotinic acid was able to prevent fibrosis via a mechanism related to its antioxidant properties and the reduction of TGF-β expression. The above findings suggested a possible role of nicotinic acid as an antifibrotic agent [78].

The data regarding vitamin B3 and UFs are very limited, therefore, available data are not highly significant in the clinical aspect and require completion. In a study by Fletcher et al. (2014), the authors studied whether NADP oxidase (NOX) was differentially expressed in the myometrium as compared with in UFs and found that the expression of NOX2 and NOX4 isoform was higher in UFs as compared with the myometrium. Interestingly, the hypoxic environment affected the expression of those enzymes, for example, increased NOX4 in the myometrium, whereas NOX1, NOX3, and NOX5 were undetectable in UFs. The conclusion was that UFs presented a unique NOX profile, which promoted a pro-oxidant state that could be responsible for their development [79]. Another study indirectly linked vitamin B3 to uterine tumors, where nicotinamide phosphoribosyltransferase (NAMPT), which catalyzes one of the steps in NAD synthesis, was involved in tumors originating from the myometrium, such as rhabdomyosarcoma (RMS) and leiomyosarcoma (LMS) [80]. Additionally, NAMPT was upregulated in some malignancies and its expression was correlated with tumor invasion, metastatic potential, and drug resistance [81]. In a study by Vora et al. (2016), the authors used tissue microarray immunohistochemistry to examine NAMPT expression in the skeletal and smooth muscle, UFs, LMSs, and RMSs. NAMPT expression was low or intermediate in the myometrium, UFs, and low-grade LMSs, whereas high-grade LMSs and most RMSs showed a high NAMPT expression. The study supported that nicotinamide-derived molecules could be differently expressed in tumors and that NAMPT was overexpressed in certain sarcomas which could correlate with tumor behavior [80]. In our opinion, vitamin B3 and its derived molecules can be of greater importance in studies concerning histological differentiation.

In summary, there are no direct studies on the use of niacin or its analogs in the area of UFs. Therefore, it seems that the vitamin itself does not play a considerable role in UF biology. While it is a difficult concept, as NAD is found all over the body and some specificity is important to begin clinical trials, more preclinical research could suggest some specific agonists which would be an interesting addition to conventional therapy in UFs, as well as in other tumors.

### 3.3. Vitamin C and Uterine Fibroids

Vitamin C (ascorbic acid) is a well-known water-soluble vitamin, mostly found in fruits and vegetables [68]. It is a widely consumed nutritional supplement and is available in many different forms, for example, tablets, capsules, and drinks. The major roles of vitamin C include antioxidant activity, as well as enzymatic reaction regulation [68]. Furthermore, it is involved in tissue repair and immune system function [82]. According to the available data, vitamin C plays a role in a variety of essential biological functions, including wound healing and collagen synthesis, which could reflect its potential role in UFs. Vitamin C deficiency in humans leads to impaired collagen synthesis contributing to the symptoms of scurvy [83].

Numerous authors have published their results on vitamin C as the panacea for various diseases. In our viewpoint, one should be careful when drawing conclusions, because a lot of the data are low quality. Therefore, some studies have expanded the understanding of vitamin C as a potential antitumor agent. However, the data are rather inconsistent [84]. A recent analysis by van Gorkom et al. (2019) showed a clinically relevant positive effect of vitamin C supplementation in cancer patients, in general, with regards to the overall survival, clinical status, and quality of life (QoL) [85]. Another study, by Nauman et al. (2019), showed that vitamin C was safe in many patient populations, with promising results in prostate cancer patients [86]. Harris et al. (2014) demonstrated that vitamin C supplementation in breast cancer patients could be associated with a reduced risk of mortality [87]. Conversely, those findings were not confirmed by other authors [88]. Nevertheless, it needs to be underlined that vitamin C therapeutic potential remains unconfirmed and controversial. According to a review by Bjelakovic et al. (2012), vitamin C supplementation did not show any effect on overall mortality [89].

There is a paucity of research on vitamin C and UFs, and therefore many directions are only hypothetical. There are interesting studies on the relationship between vitamin C and UFs in the literature. In 2011, Wise et al. published a study in which they assessed the association of the dietary intake of fruit, vegetables, and different vitamins including vitamin C [34]. No significant relationships were found among vitamin C, vitamin E, and UFs in this analysis [34]. However, Martin et al. (2011) reported a non-significant relationship between vitamin C and UFs after controlling for various variables including age, race, education, parity, age at the first birth, and age at the last birth [51]. Information concerning this issue is still insufficient. In 2017, Mesure et al. found that both TGF-β1 and ascorbic acid were able to differentiate Wharton’s jelly mesenchymal stem cells towards a smooth muscle phenotype [90]. A different study by Ji et al. (2017) showed that vitamin C deficiency could activate TGF-β signaling. The authors found that this deficiency exhibited an increased glomerular expansion and the expression of α-smooth muscle actin (α-SMA), fibronectin, and type 4 collagen, in mice. The deficiency caused the activation of Akt signaling and, consequently, induced the expression of Smad7 protein, resulting in the suppression of TGF-β/Smad signaling and ECM deposition [91]. Notably, the Akt-derived mechanism is involved in UF pathophysiology [27]. Another interesting study demonstrated that ascorbic acid could promote a TGF-β1-induced myofibroblast phenotype switch where ascorbic acid positively influenced TGF-β1-induced expression of collagen genes, α-SMA fiber formation, and the deposition of collagens type I and IV independent of Smad2/3 signaling [92]. In a more recent study, vitamin C reduced cellular recruitment, cytokine secretion and TGF-β, and collagen deposition. However, it elevated antioxidant enzymes [93]. Moreover, vitamin C has been used as an element of the medium in some experiments on UF pathophysiology. For example, vitamin C was used together with TGF-β3 to demonstrate that myometrial cells migrated into nodules containing collagen and fibronectin [94]. A recent study described vitamin C as an element of the medium for the macromolecular crowding model to alter ECM deposition in UFs [95].

Although the above data indicate conflicting results in the case of vitamin C and related fibrosis, the data also indicate that the data can differ significantly depending on the tissue. Interestingly, Heinonen et al. (2017) reported on the metabolomic profiling of UFs with *MED12* mutation and displayed the dysregulation of vitamin C metabolism [66], but the level of ascorbate itself remained unaltered. However, the authors found that the levels of dehydroascorbate, threonate, and gulonate were reduced [66]. The authors of this interesting study hypothesized that, in addition to antioxidant response, the changes in vitamin C metabolites could be connected with other cellular processes. They suggested that alterations in vitamin C metabolites could reflect adaptations in other important cellular processes including DNA and histone demethylation, as well as inflammation [66]. Recent data have suggested that ascorbate, the major form of vitamin C, could influence the activity of the genome through epigenomic process regulation [96]. Moreover, the depletion of vitamin C metabolites could be linked to the abundance of ECM in UFs, since vitamin C has been shown to be a cofactor in the synthesis of collagen [66,97]. Obviously, the data require confirmation in further research, both in laboratory and clinical settings.

It seems justified to mention a clinical aspect related to vitamin C which was emphasized in some papers. Some authors reported on the potential use of vitamin C as an addition in UF treatment. Pourmatroud et al. (2012) showed that vitamin C use during abdominal myomectomy could reduce blood loss, operation time, and days of hospitalization [98]. A more recent study by Lee et al. (2016) was performed to check that similar findings would be observed during laparoscopic myomectomy. The authors found that the operating time and the decrease in hemoglobin concentration were similar in both the study and control groups [99]. Samy et al. (2020) studied anti-hemorrhagic agents during myomectomy and found that ascorbic acid and a combination of epinephrine plus bupivacaine were the least effective pharmacological options in this indication [100]. Interestingly, endometrial thinning was effectively treated with vitamins C and E in an infertile woman undergoing myomectomy. In the described case, the patient had postoperative endometrial thinning that did not respond to hormonal treatment. The use of vitamins C and E was effective for the immediate recovery of withdrawal bleeding and gradual gain of endometrial thickness [101]. However, it is unknown whether the effect was due to the use of vitamin E instead of vitamin C, because new research showed that tocopherols can have a similar effect to estrogens [102].

Data concerning vitamin C and its influence on UF processes are currently insufficient to draw final conclusions. Therefore, because available data are limited and of low quality, more research is required to determine the influence of this vitamin on UF biology or the possibility of its use in prophylaxis or treatment.

### 3.4. Vitamin D and Uterine Fibroids

Vitamin D has been extensively studied in UF biology and therapy, and the present paper includes only the most important and recent findings. Readers who are particularly interested in the topic are encouraged to read comprehensive publications on the issue [44,103].

Vitamin D is a group of fat-soluble steroid chemical compounds which exert miscellaneous effects in the body. They control calcium-phosphate metabolism, and thus maintain the normal structure and function of the skeleton [104,105]. Traditionally, vitamin D belongs to vitamins, but it also performs the function of a prohormone. Vitamin D can occur in several forms as follows: vitamin D1, calciferol (occurs most commonly in fish oils); vitamin D2, ergocalciferol (occurs in plants); and vitamin D3, cholecalciferol (produced in the skin) [105,106].

Reduced levels of vitamin D have been confirmed in several gynecological and obstetric pathologies, for example, infertility, polycystic ovary syndrome, and premature delivery [107]. Abnormal levels of vitamin D in the blood serum are also currently viewed to be a potential risk factor of UF development [44]. Ethnicity is also involved in such risk disparity [3,108], since lower concentrations of vitamin D are more commonly reported in dark-skinned women (especially of African American origin). An epidemiologic study concerning women living in the USA revealed that approximately 80% of African Americans had vitamin D deficiency as compared with a group of white women in whom abnormal vitamin D concentrations were found only in 20% of the group [109,110]. As found by Baird et al. (2003), a similar number of African American women was also diagnosed with UFs and these tumors developed in black women at earlier ages than in white women [3].

Vitamin D has been one of the leading contributors to the current theory of UF pathogenesis, for approximately a decade [103]. Recent studies have shown a negative correlation between vitamin D levels in blood serum and the presence of UFs [108,111,112]. Lower vitamin D levels in blood serum, in women, were more commonly associated with UFs with the correlation being present regardless of ethnicity [108]. The odds of UF occurrence in patients with sufficient vitamin D were estimated to be lower by 32% as compared with patients with vitamin D insufficiency. Interestingly, the authors also found that sun exposure was associated with a reduced risk of UF occurrence [108]. According to other authors, women diagnosed with infertility and concomitant UFs had significantly lower levels of vitamin D than infertile patients without UFs (18.0 ± 7.7 and 20.8 ± 11.1 ng/mL, respectively). The number of subjects with 25(OH)D level lower than 10 ng/mL equaled 15% in the study group and 7% in the controls [112]. The most recent of the three main studies concerning the correlation of vitamin D and UFs showed that vitamin D concentration in blood serum was lower in a group of patients with symptomatic UFs than in a control group [111]. The study included 154 premenopausal women, out of whom 50 women were UF-free controls. The authors found an inverse correlation between serum 25(OH)D levels and the total UF volume. This means that patients with larger UF volumes had lower serum vitamin D levels [111]. Similar conclusions were made by researchers from Italy [113] and India [114,115]. In both studies from India, the patients with UFs had much lower 25(OH)D serum levels as compared with the UF-negative controls [114,115]. In 2018, authors from Turkey found that vitamin D levels were lower in UF-positive patients, but they found no correlation between those levels and the volume, location, and number of UFs [116]. Lower serum levels of vitamin D were also highly prevalent in patients with UFs, in a recent study from China, performed on a total of 546 participants. Serum calcium levels were found to be in the normal range in both groups [117]. Another study also indicated 25(OH)D as a risk factor for UFs among obesity, positive family history for UFs, and higher transforming growth factor β3 [118]. There was only one study that found no association between low vitamin D levels and the occurrence of UFs within the selected population. The study was conducted by Mitro and Zota in 3590 patients, who found that insufficient 25(OH)D serum concentration was not associated with UF occurrence in a population-based analyses. However, an analysis corrected for misclassification suggested such an association in white women [119].

Laboratory studies have been conducted for a better understanding of the vitamin D effect on UF growth. One study demonstrated that active 1, 25(OH)D inhibited the growth of UF cells collected in women who had undergone a hysterectomy in a dose-dependent manner [120]. Further research showed an antifibrotic effect of vitamin D on UFs via the reduction of TGF-β3-induced ECM protein expression including fibronectin and collagens in UF cells [121]. Sharan et al. (2011) indicated that vitamin D inhibited UF cell growth through the downregulation of proliferating cell nuclear antigen and cyclin-dependent kinase 1, as well as the inhibition of catechol-O-methyltransferase expression and activity [122]. Subsequently, in vivo research showed that vitamin D decreased the size of UFs in Eker rat model [123]. Moreover, vitamin D regulated the expression and activity of matrix metalloproteinases (MMPs), which are enzymes that play a role in ECM remodeling [124]. In 2015, the same group showed that vitamin D presented potent antiestrogenic and antiprogesteronic activities, where vitamin D treatment significantly reduced the expression of estrogen and progesterone receptors through the induction of VDR expression [125]. Vitamin D also inhibited Wnt/β-catenin activation in UFs with *MED12* gene mutations [126,127]. In 2018, further research proved that vitamin D deficiency could enhance DNA damage in the myometrium toward the UF phenotype [128]. An interesting study by Ali et al. (2019) also showed that vitamin D suppressed UFs through targeting different networks in DNA repair [129]. The latest research concerning UFs and vitamin D has focused on the role of vitamin D in the Wnt/β-catenin-dependent pathway. Corachan et al. (2019) stated that vitamin D worked as an antiproliferative compound in UFs through cell growth arrest and Wnt/β-catenin pathway inhibition [130]. Vitamin D induced a significant tumor volume reduction through reduced cell proliferation, the reduction of TGF-β3 expression, and apoptosis induction [131].

Moreover, some data are available on the use of vitamin D in humans. Italian researchers found that vitamin D could affect small UFs [113]. In 2019, Hajhashemi et al. presented their results on the use of vitamin D for 10 weeks in patients with UFs. Their results showed that UF size significantly decreased in the group that used vitamin D as compared with a placebo group [132]. Arjeh et al. (2020) recently tested high-dose vitamin D in a randomized clinical trial for 12 weeks. The treatment did not change the volume of lesions, but it inhibited their further growth, while the volumes of UFs in the placebo group increased [133]. Furthermore, some studies were conducted on the potential use of vitamin D together with other anti-UF compounds. This is discussed later in this manuscript.

Several genes are involved in vitamin D metabolism and several single nucleotide polymorphisms (SNPs) are associated with 25(OH)D concentrations. Wise et al. (2014) investigated the incidence of UFs in relation to polymorphism in genes involved in vitamin D metabolism. Two single nucleotide polymorphisms were found which were significantly associated with UFs. One was near *DHCR7* and the other was in *ASIP*.

To sum up, vitamin D could be a potential inexpensive and safe agent for both the prevention and treatment of UFs. We suggest that future strategies for the fight against vitamin D deficiency in women with UFs should be implemented in daily medical practice, for example, by means of tests performed in patients in whom conservative treatment has been attempted. It has been corroborated by new research, but to fully explore the clinical utility of vitamin D in UFs, we still need a multicenter randomized placebo-controlled clinical trial to confirm the supportive evidence from preclinical and limited pilot clinical studies [103,134]. Research facilitating the identification of patients in whom such testing could be of the highest clinical value would be particularly important. It would also be interesting to determine safe and the most effective doses.

### 3.5. Vitamin E and Uterine Fibroids

Vitamin E is a group of fat-soluble organic chemical compounds [135,136]. Vitamin E is essential for the appropriate structure of biological membranes [136,137]. According to some studies, vitamin E deficiency can have a negative impact on body functioning, including disorders of fatty acid conversion, fertility disorders, miscarriages, the abnormal function of the cardiovascular system, nervous system, and skin [138,139]. Conversely, an excess of vitamin E in the diet is also undesirable, as it can be associated with the following manifestations: visual disorders, headaches, gastrointestinal problems, and weakness [140]. According to recent trials, vitamin E supplementation does not improve health or mortality rates and is not effective in disease prevention [141]. Interestingly, it has even been found to be a possible risk factor for prostate cancer in men [142]. α-Tocopherol is considered to be the most active form of vitamin E and the second most common form of vitamin E in the diet [143]. The biological activity of vitamin E is dependent upon α-tocopherol transfer protein (α-TTP) regulation which recognizes and moves α-tocopherol to different tissues [143,144]. Vitamin E is a highly potent antioxidant that inhibits the production of reactive oxygen species upon fat oxidation and during the propagation of free radical reactions [145].

As for the correlation between vitamin E and UF development, Wise et al. (2011) who followed 22,583 premenopausal women [34] and Martin et al.’s (2011) study in 887 women [51] revealed no significant relationship between vitamin E and UFs. A study by Ciebiera et al. (2018) demonstrated a correlation between elevated concentrations of one of the vitamin E vitamers (α-tocopherol) in the blood serum and UF occurrence in Caucasian women, but the study was conducted in a much smaller group than the previous studies [146]. Therefore, the positive influence of vitamin E could depend on particular gene polymorphisms. According to available data, UFs present impaired antioxidant activity [147]. UFs have also been found to present lower activities of superoxide dismutase and catalase as compared with the myometrium [147,148]. According to Fletcher et al. (2017), enhanced oxidative stress was associated with decreased apoptosis and it could be connected with the transformation of healthy myometrial cell towards fibroid-like cells [147]. In such a case, vitamin E could serve as a protective factor through free radical removal.

Data concerning vitamin E and UFs are insufficient to draw final conclusions. Regrettably, some available literature only offered hypotheses. Therefore, similar pathophysiological models should be analyzed. Studies connecting fibrosis and vitamin E were performed in nonalcoholic hepatosteatosis, where vitamin E improved fibrosis scores [149]. Although these results are still controversial, Di Sario et al. (2007) stated that increased serum levels of tocopherols following vitamin E supplementation did not necessarily result in liver protection [150]. Mechanistically, tocopherols can inhibit the growth of cultured cancer cells through several effects, such as trapping free radicals, downregulating cyclins, and preventing proliferation in this way [145,151]. Moreover, vitamin E can affect gene expression and regulate enzymes activity such as protein kinase C, which is important in smooth muscle growth [152,153], where vitamin E deactivates protein kinase C and inhibits the growth of smooth muscle cells [154,155]. In a study by Gysin et al. (2002), α-tocopherol inhibited the production of protein kinase C and collagenase [152].

In the pathophysiology of UFs, it is interesting that vitamin E contains structural determinants that make it a possible ligand for estrogen receptors (ERs) [102,156] and it can be described as a specific phytoestrogen [102,146]. A study conducted in postmenopausal women showed that vitamin E reduced the symptoms associated with the lack of estrogens, for ecample, hot flashes or sweating [157]. Furthermore, three months of vitamin E supplementation in women suffering from implantation failure resulted in beneficial effects on endometrial measurements [158]. In this case, it would be interesting to conduct studies in postmenopausal women who supplement vitamin E in order to check its effect on the endometrium, bleeding profile, and the size of possible UFs. Vitamin E also affects the release of various growth factors which can be important in the pathophysiology of UFs, for example, epidermal growth factor (EGF) [159]. Such effects of tocopherols on endometrial growth could be attributed to only the antioxidant properties which stabilized the tissue [160]. However, the phytoestrogen activity could also be contributing [146].

Because the data have suggested that higher concentrations of vitamin E could be a risk factor in some patients [146], considering the analogs could be a valid alternative. According to Young et al. (2004), vitamin E succinate (a vitamin E analog) reduced UF cell number and induced cell death [161]. Notably, this succinate ester is a much more powerful antitumor agent than other tocopherols in apoptosis induction [162]. Zhang et al. (2002) also found that vitamin E succinate ester could act as a steroid hormone signaling inhibitor [163].

To summarize, according to the limited available reports, the influence of vitamin E on UFs remains unclear, but there are some factors which could be of interest in UF development and be a part of therapy in the future.

### 3.6. Vitamin K and Uterine Fibroids

Literature data on the direct relation of vitamin K and UF are rare and rather indirect. Some papers tackled this issue but, regrettably, these papers mostly offered hypotheses. Vitamin K is a group of organic chemical compounds which are derivatives of 2-methyl-1,4-naphthoquinone. Vitamin K includes the following two natural vitamers: vitamin K1 (phylloquinone) and vitamin K2 (menaquinone). Moreover, some synthetic vitamers are also available, such as vitamin K3 (menadione). Gut microbiome can convert K1 into different vitamers of vitamin K2 [164]. Vitamin K is known in medicine mostly because it is fundamental in blood clotting processes. Moreover, it also participates in skeletal system metabolism and affects the immune system [165]. Vitamin K is involved in the carboxylation of glutamate residues in proteins to form gamma-carboxyglutamate (Gla) residues which bind and activate calcium ions. These residues are situated within specific protein domains called Gla domains [166], which play an extremely important role in blood coagulation processes [166,167], as well as bone metabolism [168,169,170]. Gla domains were studied in vascular pathophysiology, especially in the area of growth arrest-specific protein 6 (Gas6) which plays a role in cell growth (in the endothelium and smooth muscle cells), apoptosis, phagocytosis [171], with the last one being possibly connected with UFs. According to a study by Sun et al. (2003), the levels of Gas6 mRNAs were significantly higher in UFs than in the healthy myometrium. The authors suggested that Gas6 signal transduction could be related to UF growth and cause aberrant stimulation [172]. Varnum et al. (1995) stated that the transforming activity of Axl and its receptor could drive cellular proliferation in response to an appropriate signal, namely a ligand that activated the receptor and this receptor was stimulated by the vitamin K-dependent protein encoded by the Gas6 gene [173].

Because there are limited studies concerning vitamin K in UFs, we explored some of the possible mechanisms using cancer models that considered its anti-inflammatory, antioxidative, and anti-carcinogenic properties [174]. Xv et al. (2018) concluded that vitamin K2 could positively inhibit the growth of cancer cells. The authors emphasized that a combination treatment of vitamin K2 and chemotherapeutics could lead to better results with fewer side effects. However, much more data are necessary to draw final conclusions [175]. Zhong et al. (2012) concluded that vitamin K2 significantly improved one-year overall survival in patients with liver cancer [176]. Other studies have shown that vitamin K could influence common tumor pathways and induce cell cycle arrest [175]. For example, according to Sibayama-Imazu et al., vitamin K2 could activate Janus kinases to phosphorylate orphan receptor TR3 [177]. It has also been reported that vitamin K could influence extracellular signal-regulated kinase phosphorylation and, subsequently, change the activation of mitogen-activated kinases, which influence apoptosis in some types of cancer [178]. Vitamin K has an important anti-inflammatory role [179,180] which has been confirmed mainly in animal studies. According to Ohsaki et al. (2006), vitamin K suppressed lipopolysaccharide (LPS)-induced inflammation [181]. A different study demonstrated that interleukin 6 production in the LPS-induced fibroblasts was inhibited by vitamin K [182]. In regards to human studies, Shea et al. (2008) found that higher vitamin K use could be associated with the lower concentrations of proinflammatory markers [180].

Some authors have also tried to determine whether the use of vitamin K could prevent UF development and growth in vitro and in animals. Chlebowski et al. (1985) showed that vitamin K was effective in inhibiting the growth of tumor cells [183], whereas Nestor et al. (1991) suggested that vitamin K did not affect UF and LMS cells and its high intake did not increase the circulating levels or the activity or reduce the risk of UF occurrence in poultry [184]. The authors suggested that vitamin K was not be as effective in humans and animals as in cell cultures, and there could be an influence by cytokines, growth factors, or hormones that negatively affected its activity [184,185].

The above data concerning the possible influence of vitamin K on UFs need some supplementation, but it seems highly possible that the influence on UF tumors is rather negligible.

We summarize the available data about vitamins in uterine fibroids in Table 1.

### 3.7. New Trends and Possible Future Role of Vitamins in Uterine Fibroids Management

Women who do not want to undergo a hysterectomy because of reproductive plans or other various reasons are not deprived of other effective treatment modalities [10,13,14]. Nowadays, the first-line treatment of UFs usually involves pharmaceutical treatment which leads to the resolution of symptoms. According to a number of guidelines, the first-line treatment includes hormonal methods to reduce abnormal uterine bleeding or to prepare the patient for definitive treatment [13,187]. Drugs which have proven to be particularly effective in UF treatment are GnRH analogs and selective progesterone receptor modulators (SPRMs) [13]. The selection of drugs is not extensive, especially if compared to the scope of the problem, due to the number of patients requiring treatment, and the costs generated in case of those lesions [12]. GnRH analogs faced the chance of becoming a new trend in this indication, especially after they were introduced into the market in the oral form, for example, elagolix [188] or linzagolix [189]. An increasing number of studies published recently have shown their beneficial influence on UFs and UF-related manifestations [190,191].

The most recent attempts at developing an inexpensive, safe, and effective drug to provide UF prophylaxis and treatment are at an early stage and it is unknown whether they will be successful [7]. Currently, the therapies used are rather short term to avoid the risk associated with long-term hormonal treatment and possible long-term negative effects in the body [192]. Alternative solutions are necessary in UF patients, because not all of them accept hormonal or operative treatment. Results that have been obtained recently have allowed us to assume that substances included in green tea, vitamin D, or elagolix could turn out to be preparations used long term with minimal adverse effects [42]. Similar concepts have been considered in the research aiming at developing a specific method of UF prophylaxis in women who are at an increased risk of the development of those tumors [7,44].

Regardless of the developing market of oral GnRH analogs, other compounds should also be considered, both as basic and accessory therapy options [42]. Global tendencies show that future research may tend to concentrate on the possibilities for the use of natural compounds in various therapies. Such a trend seems to be strong in terms of UFs and some of the ideas have been confirmed in trials [42]. In new studies, the use of vitamin D and its effects on UFs have shown that microelements could be key factors in the development of some pathologies [44]. Most of the studies corroborating the effectiveness of this therapy in humans are valuable [113,133] and should be promoted among physicians. Importantly, subsequent steps should involve the adjustment of doses and testing on the largest groups possible. New randomized trials constitute another step in the research on vitamin D and other drugs. However, it is also necessary to conduct research on the synergistic associations between various compounds, such as the synergy which has already been confirmed between vitamin D and epigallocatechin gallate [193], or between vitamin D and ulipristal acetate [194]. In a recent study by Porcaro et al., UF volumes decreased by 34.7% in the treated group; however, they increased by 6.9% in the controls. An improvement in selected symptoms in women treated with the use of vitamin D, EGCG, and vitamin B6 was reported. Therefore, the authors concluded that it could be an optimal approach in some patients [193]; however, it may be too early to draw final conclusions. Nevertheless, research which led to such conclusions should be continued. Another synergism was identified for vitamin D and ulipristal acetate. Ali et al. (2020) demonstrated that a significant dose- and time-dependent growth inhibition effect of ulipristal acetate and vitamin D combinations occurred in an experimental model as compared with the untreated cells on days two and four [129]. In this case, results of human studies are practically unavailable. The simultaneous use of the abovementioned compounds was described in humans in two cases, which made it possible to conclude that ulipristal acetate and vitamin D presented good clinical effectiveness as anti-UF agents sharing synergistic antifibroid properties. We believe that new compounds that could be involved in such synergies should be investigated. Seemingly, the data justify the inclusion of vitamin D concentration measurements in the clinical proceedings in UF patients or in risk groups. The use of vitamin D analogs is a valid direction of research associated with UFs and vitamin D.

Since vitamins are extensively consumed worldwide, both UF patients and physicians could wonder about their possible effect against UFs. However, such strong evidence has not been obtained, except for vitamin D. We presented an example of important pathways associated with vitamin A and UFs which contributed to the pathophysiology of the tumors as a very important signal transduction pathway [27]. The use of vitamin A in UF treatment seems to be a new promising direction which requires further research [64,65]. In our viewpoint, research in this area should comprise different forms of vitamin A, as well as provitamin A. It appears that various vitamin A isomers would be useful in UF treatment, as has been observed in acne problems [195]. It would be interesting to study a group of patients using isotretinoin as dermal lesion treatment to assess the possible changes within the reproductive organs and lesions concomitant with UFs. Another interesting issue in UF treatment is related to drugs altering vitamin A metabolism [56], as has been demonstrated in the case of liarozole [64]. However, because only solitary studies are available and data are too scarce to draw conclusions, we are looking forward to other results.

Another, but maybe a less interesting, area of research on vitamins and UFs is associated with vitamin B3. The data are scarce, but some conclusions and hypotheses are intriguing. The vitamin itself can have very low or even no influence, but the molecules in which it occurs, or those which need this vitamin to be produced, are significant in UF research. For example, a group of sirtuin proteins is an interesting starting point for possible future therapies. Because of the NAD-related correlations between sirtuins and fibrosis it appears that drugs altering NAD metabolism could be successfully used in UF treatment. Such drugs have recently been introduced in other conditions involving fibrosis, for example, in dermatoses or in the lungs [196]. The use of NAMPT [80] (e.g., in the differentiation of tumors such as UFs and LMS) is another interesting issue which is also indirectly related to vitamin B3 and UFs. Although there has been considerable progress in the differentiation of such pathologies, some lesions are still very difficult to distinguish [2,197]. It is possible that the use of such new markers could help in the effective differentiation of lesions, which would provide the patient with the selection of a suitable therapy.

Vitamin C can also have an influence on UFs, especially with revealed changes in metabolomic profiling [66] or epigenetic control [96]. Seemingly, some mechanisms associated with this vitamin could be of key importance in the development of those tumors. They may not be associated with all of them, but with those having specific mutations, for example, *MED12* mutants. Vitamin C does not seem to be associated with the risk of developing UFs, but future research could comprise such aspects such as its possible, various analogs in UF cotreatment. Examples of using vitamin C and its analogs and derivatives are found in dermatology [198], and therefore some data could be referred to regarding this discussed matter. It ha been demonstrated that the use of vitamin C in the treatment of intraoperative bleeding was rather experimental and presented no considerable clinical effect [100].

In addition, the situation regarding vitamin E is not fully clear. Studies conducted on large groups have shown that this vitamin has no influence on UF risk [34,51]. However, some groups could be predisposed to an increased risk for the development of those tumors due to high concentrations of some vitamers [146]. Particular attention should be paid to the abovementioned estrogen component associated with vitamin E [102], because it could be the reason for UF growth in some patients, for example, with hypersensitivity. Vitamin E analogs are other noteworthy issues to be considered [161]. There have been no studies conducted in this area for many years. However, the substances could perform some modulatory functions, including ones influencing estrogen receptors.

We should emphasize that surgical methods and drug therapies, with their effectiveness confirmed in subsequent clinical trials, are most important in the current and near future perspective. The data presented above comprise almost the whole knowledge concerning vitamins and aim at indicating potential directions of development and possible research to be conducted in the near future. Importantly, we should remember in which biological pathways vitamins play a role and how such knowledge could be implemented. It is also important that certain vitamins are introduced as an adjuvant therapy for already-existing therapies or as a form of prophylaxis following confirmation obtained during research. At this point, we want to emphasize the idea of using combined drug treatment in UF therapy [29,194]. Some of the above discussed vitamins and related data seem to be suitable for such research. This could be true for vitamin A and vitamin A analogs, vitamin E analogs, and, particularly, for vitamin D for which this hypothesis was partially confirmed [193,194,199]. Individualized drug selection for patients after the preliminary verification of various factors, such as the ethnicity, manifestations, concomitant factors, or diseases, is an idea for the future, as the use of a single drug should not be generalized for every patient [200]. It is important to search for a synergistic or antagonistic potential between drugs in UF treatment and combine drugs for an optimal effect.

## 4. Conclusions

The presented comprehensive review showed that some vitamins are involved in the biology and pathophysiology of UFs. The review demonstrated that vitamins A and D deserve special attention due to the abundance of available studies and evidence regarding their influence on the biology and treatment of UF tumors. Therefore, they could provide new directions in UF pharmacological prevention or cotreatment. The literature concerning vitamin D is extensive and the effects exerted by this vitamin seem to be confirmed. Recent studies have concentrated on the effectiveness of various doses of vitamin D in UF treatment in randomized clinical trials. Secondly, it is worth noting that vitamins B3, C and E have not been as widely studied as the abovementioned ones. Therefore, no specific conclusions can be drawn on their application in UFs. Obviously, the research is still incomplete. The influence of vitamin K on UFs is rather negligible. Other vitamins do not seem to be correlated with UFs, or the correlations have not been determined so far.

## Figures and Tables

**Table 1:** The established and potential role of vitamins in uterine fibroids (the italics indicates possible role).

**Vitamin A**	Role in signal transduction in uterine fibroids [52,55,67]
	Cell proliferation control [54,60,65]
	Extracellular matrix formation control [60]
	Antifibrotic effect [60,63]
	Tumor growth control [65]
	*Potential protective role* [34]
**Vitamin B3**	Coenzyme in uterine fibroid biology [68,79]
	*Potential anti-inflammatory effect* [76]
	*Potential role in oncogenesis* [80]
**Vitamin C**	Cell differentiation control [90]
	Dysregulation of vitamin C metabolism in MED12 mutants [66]
	Antioxidant effect [68]
	*Potential antifibrotic effect* [92,93]
**Vitamin D**	Role in signal transduction in uterine fibroids [122,131]
	Cell proliferation control [130]
	Extracellular matrix formation control [186]
	Antifibrotic effect [121]
	Tumor growth control [120,123]
	*Potential protective role* [133]
**Vitamin E**	Antioxidant effect [145,147]
	*Phytoestrogen, potential role in tumor development and growth* [102,146]
**Vitamin K**	*Potential anti-inflammatory effect* [179,180]
	*Potential antifibrotic effect* [182]

## Abbreviations

25(OH)D	25-hydroxyvitamin D
BMI	body mass index
ECM	extracellular matrix
EGF	epidermal growth factor
ERK	extracellular signal-regulated kinase
FGF	fibroblast growth factor
Gas6	growth arrest-specific protein 6
Gla	gamma-carboxyglutamate
GnRH	gonadotropin-releasing hormone
GPER	G protein-coupled estrogen receptor
LMS	leiomyosarcoma
MED12	mediator complex subunit 12
MMP	matrix metalloproteinase
mRNA	messenger RNA
NAD	nicotinamide adenine dinucleotide
NADP	nicotinamide adenine dinucleotide phosphate
NAMPT	nicotinamide phosphoribosyltransferase
NF-κB	nuclear factor kappa-light-chain-enhancer of activated B cells
NIACR1	niacin receptor 1
NOX	NADP oxidase
PI3K	phosphoinositide 3-kinase
PPAR	peroxisome proliferator-activated receptor
QoL	quality of life
RA	retinoic acid
RMS	rhabdomyosarcoma
RXR	retinoid X receptor
SPRM	selective progesterone receptor modulator
TGF-β	transforming growth factor β
TNF-α	tumor necrosis factor α
UF	uterine fibroid
VDR	vitamin D receptor
VEGF	vascular endothelial growth factor
Wnt	wingless type
α-SMA	α-smooth muscle actin
α-TTP	α-tocopherol transfer protein
